# Supplementary material for: Learning environment-specific learning rates
Source: PLoS Comput Biol. 2024 Mar 22;20(3):e1011978. doi: 10.1371/journal.pcbi.1011978 (PMC10990245; doi:10.1371/journal.pcbi.1011978)
Supplement: Attachment — Submitted filename: Response_Letter.pdf [file pcbi.1011978.s001.pdf]

FACULTY OF PSYCHOLOGY AND EDUCATIONAL SCIENCES

DEPARTMENT OF EXPERIMENTAL PSYCHOLOGY

COGNITIVE COMPUTATIONAL NEUROSCIENCE GROUP

Dear Prof. Dr. Lusha Zhu and Prof. Dr. Marieke van Vugt,

We would like to express our gratitude for considering our manuscript "Learning environment-specific learning rates" for publication. We appreciate the time and effort you have invested in evaluating our submission.

We are also grateful to the reviewers for providing constructive feedback that enabled us to significantly improve the quality of our manuscript. We have carefully considered the reviewers' suggestions and revised the manuscript to address their concerns. Please find our detailed responses below.

We hope the revised manuscript is now suitable for publication in *PLOS Computational Biology*.

Sincerely,

Jonas Simoens, Tom Verguts, and Senne Braem

GHENT UNIVERSITY

Reviewer 1:

In this study, the authors investigated the relationship between learning rates and environment volatility in reinforcement learning tasks. Across 3 different experiments and variations of dual bandit tasks with different volatility conditions, they show that reinforcement learning models implementing environment specific learning rates fitted better the data compared to models that were myopic to volatility conditions. They found that subjects used overall higher learning rates in volatile environments and were able to switch between high and low learning rates depending on the environment they interacted with.

The question investigated in the present study (i.e., Can people learn to use environment specific learning rates adapted to different volatility conditions?) is interesting and various experiments and analyses have been implemented to answer that question. However, analyses and results are not entirely convincing and do not answer satisfactorily the aforementioned question. In its current version, the manuscript also lacks clarity and details across all sections. I introduce below a series of major and minor comments.

Major Comments

In the current version of the manuscript, the authors provide a parameter recovery analysis in the methods section. However, no model recovery analysis is provided. This analysis is of prime importance insofar as the model comparison presented in the results section constitutes the primary result of the study. This would confirm that the model selection procedure used to fit the data in this particular task is actually able to distinguish the different models. Could the authors provide such an analysis?

We now added a model recovery analysis which indicates that our experimental design and our model selection procedure allow for a reliable selection of the model that fits the data best. The model recovery analysis can be found on page 30:

> "To ensure that the experimental design and model selection procedure would allow for the reliable selection of the model fitting the data best, we performed model recovery simulations. Specifically, using the learning phase described above, we simulated 10 datasets with each of the six models described above. For all model

parameters we used the estimated posterior means from Experiment 1 as the means of the group-level distribution. These means were 0.4 and 0.6 for stable and volatile learning rates, respectively, in all three environment-specific models; 3.5 and 4.5 for stable and volatile inverse temperatures, respectively, in all three environment-specific models; 0 for the stable as well as the volatile higher-order learning rate in the variable learning rate model; 0.5 for learning rates in all non-environment-specific models; 4 for inverse temperatures in all non-environment specific models; and 0 for the higher-order learning rate in the variable learning rate model. We then randomly sampled 36 (i.e., the number of participants we used in Experiment 1) values from each group-level distribution with the relevant mean and an SD of 0.2 for learning rates in all models, 2 for inverse temperatures in all models, and 0.1 for higher-order learning rates in the variable learning rate models. For each model, we then simulated a dataset using these parameter values and two blocks of 120 trials per participant (i.e., the number of trials we used in Experiment 1) and fitted all models to this simulated dataset using the model estimation procedure described above.

Finally, using the model selection procedure described above, we tested for each simulated dataset which model fitted it best. As can be seen in Table 6, all models can be reliably recovered. In experiments 2 and 3, we increased the sample size in terms of participants as well as trials per participant. Thus, here, model recovery rates were undoubtedly at least as high."

Table 6

Model recovery simulation results

Model	1	2	3	4	5	6
Environment-specific dual learning rate model (1)	0.9	0.1	0	0	0	0
Environment-specific single learning rate model (2)	0	1	0	0	0	0
Environment-specific variable learning rate model (3)	0	0	1	0	0	0
Non-environment-specific dual learning rate model (4)	0	0	0	1	0	0
Non-environment-specific single learning rate model (5)	0	0	0	0	1	0
Non-environment-specific variable learning rate model (6)	0	0	0	0	0	1

Note. Presented here are the probabilities that a given model (column) fitted a dataset generated with a given model (row) best.

The temperature parameter is totally absent in the current version of the results. This parameter is an essential piece in analyses such as those presented, as a difference in Q-values has a radically different impact on choice depending on the temperature value. Considering the importance of this parameter, the results section and/or the figures should include a comparison of the temperature parameters in the two volatility conditions.

We indeed forgot to report the temperature analyses, as our main focus was on the learning rates. However, we agree this information is important, and now added a visual as well as a statistical comparison of inverse temperatures between both environments for each experiment. For example, for Experiment 2, these comparisons can be found on page 11:

> "The posterior probability that inverse temperatures were lower in the volatile than in the stable environment was 0.927."

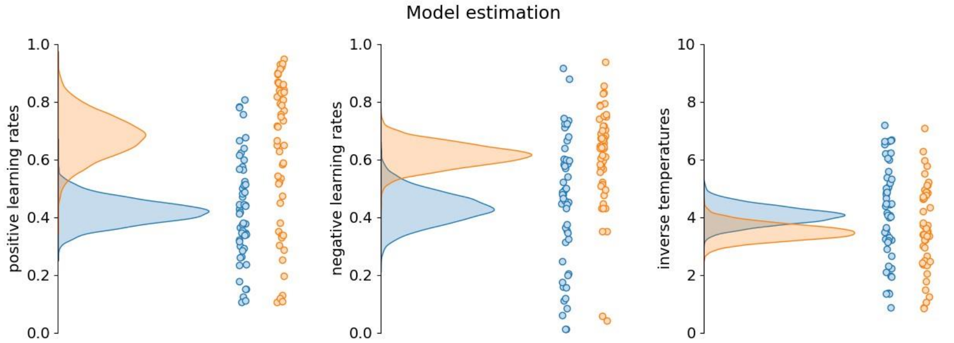

The results for Experiment 1 (page 9) and Experiment 3 (page 15) are very similar.

Authors found that models with different learning rates for positive and negative prediction errors fit better the data. Authors state that "This is likely because negative learning rates are more important than positive learning rates when adapting to changed reward contingencies." However they give no details about the mechanism underlying this result, and the fitted learning rates (for positive and negative prediction errors) seem equal to one another (Figure 3). Could the authors give details about these mechanisms? It would be helpful to see if a potential asymmetry in learning rates differs between the two volatility conditions and across periods (experiment 2), and could explain partly the results observed.

If there is no interaction between learning rates asymmetry and volatility conditions, it would then be interesting to see how would perform a model with only two learning rates (for positive and negative PEs) and a single scaling parameter (adjusting learning rates between the two volatility conditions) compared to the winning model presented here.

Our statement that negative learning rates might be more important than positive learning rates was mostly inspired by our optimality analyses. However, we agree that this could be motivated better and more clearly, and therefore added an explanation of why we believe environment-specific negative learning rates are more important than environment-specific positive learning rates on page 9:

> "This effect is more pronounced for negative learning rates (i.e., learning from negative prediction errors). More specifically, in the stable environment, lower negative learning rates tend to result in higher reward rates, while positive learning rates have less effect on reward rate. In the volatile environment, higher negative as well as positive learning rates tend to result in higher reward rates, but this effect is still more pronounced for negative than for positive learning rates. This is likely because negative learning rates are more important than positive learning rates when adapting to changed reward contingencies. That is, since one is likely to experience a lot of negative prediction errors immediately following a reward contingency change, a higher negative learning rate will enable one to adapt faster to this change. Crucially, here, reward contingency changes exclusively occurred in the volatile environment."

As suggested by the reviewer, we also tested whether the asymmetry in learning rates differed between the two volatility conditions, but this was not the case. We further tested whether a model with only two learning rates and a single scaling parameter fitted the data better than the environment-specific dual learning rate model, but this was also not the case. However, for each experiment, we now added a figure in which participants' estimated positive and negative learning rates are plotted over the reward rate simulation results for the dual learning rate model. These figures show that, on an individual level, there are substantial differences in the extent to and direction in which positive and negative learning rates differ

from each other. This explains why, on a group level, they do not differ from each other. We also added this explanation to the manuscript on page 9. For example, for Experiment 2, this figure can be found on page 13:

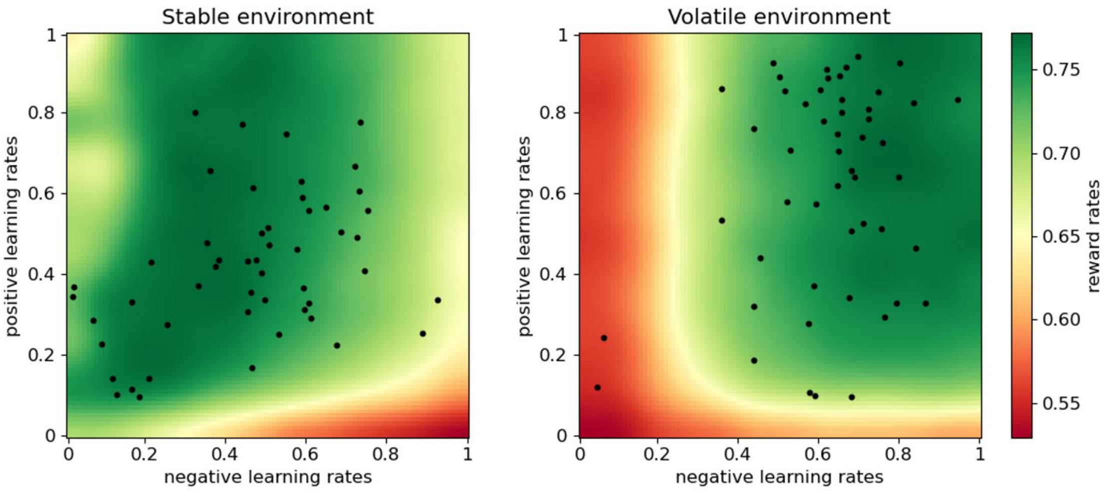

The figures look very similar for Experiment 1 (page 10) and Experiment 3 (page 16).

From figures 3-5, it seems that subjects have very different learning rates. How these differences relate to the current results? Is there a group that adapts to the volatility of the environment and another one that does not? This is also important in regards to the message of the manuscript where it is stated that people use "optimal" learning rates in both environments, whereas the range of learning rate values of subjects in the experiments is extremely large.

In line with our hypothesis, averaged over participants, learning rates are higher in the volatile than in the stable environment, which is a difference in the direction of optimal learning rates. However, there is substantial variance in participants' learning rates, and, thus, in their proximity to optimality. Therefore, in our revised manuscript, we removed all references to participants using "optimal" learning rates.

Do people switch back and forth between two pairs of learning rates according to the environment they interact with? On average, learning rates are found to be higher in the high

volatility condition but there is little evidence of a switch back and forth between two pairs of "optimal" learning rates. The test phase from experiment 3 shows that subjects adapt quickly (over a block) to the volatility of the environment. Results from experiments 2 and 3 could then be interpreted as a series of adaptations to high and low volatility environments. The hypothesis of an increase in the difference between learning rates over blocks found statistical evidence in experiment 3 only. The authors state that subjects learn to use "optimal" learning rates over blocks but do not provide any measures indicating that learning rates are indeed more "reward maximising" in the last block compared to the first one.

The test phase in Experiment 3 indeed suggests that participants quickly adapt their learning rates to the volatility of their environment. This could explain why learning rates are already higher in the volatile than in the stable environment in the first block of both Experiment 2 and 3. This, in turn, might explain why this difference did not change much more over the course of the later blocks. It is very unlikely, however, that participants continuously adapted one pair of learning rates to the environment they were in, since they randomly switched back and forth between each environment on a trial-by-trial basis. This means that they never had more than a couple of trials in a row (and often only one trial) in the same environment before switching to the other environment again, which is not enough to infer the volatility level and corresponding learning rates. It is more likely that they learned this for both environments simultaneously, switching back and forth between the appropriate learning rates. To clarify this in the manuscript, we added on page 4 that:

> "Crucially, participants randomly alternated between both casinos, made recognisable by pictures preceding each trial as well as the location and colour of the slot machines, on a trial-by-trial basis (see Figure 1A). As a result, the number of consecutive trials that participants received the same casino was 1 for 50% of the time, 2 for 25.07% of the time, 3 for 12.37% of the time, 4 for 6.4 % of the time, 5 for 3.04% of the time, 6 for 1.49% of the time, and anything over 6 for less than 1% of the time."

In response to a previous comment, we already removed all references to participants using "optimal" learning rates. However, for both Experiment 2 and 3, we added an analysis that

demonstrates that participants acquired higher reward rates in the last compared to the first block of the learning phase. For example, for Experiment 2, this analysis can be found on page 14:

> "In line with our reward rate simulations (Figure 3B), which suggest that using higher learning rates in the volatile than in the stable environment is more reward maximising, reward rates were significantly higher in the fourth compared to the first block ($t(50) = 2.239$, $p = .015$)."

This result was very similar for Experiment 3 (page 17).

Minor Comments

Concerning the simulations performed with the winning model, authors state that they used the " inverse temperatures fixed to the optimal value for each environment". It would be very helpful (even essential) for the reader's understanding to know what are the values of these parameters and if they differ, why this is the case.

On page 9 of the manuscript, we added that:

> "We only visualised the interactions between positive and negative learning rates, with inverse temperatures fixed to 4, the average observed inverse temperature. However, the results look almost identical with higher, and therefore closer to optimal, inverse temperatures."

Even though we can guess that the winning model has 6 parameters, 4 learning rates and 2 temperatures, it is hard to be sure of that as it is not written clearly in the manuscript. I would suggest to detail the list of parameters used for each model, summarised in a table for instance.

We agree this helps keeping track of the different models. Therefore, we added the following table to the manuscript on page 28:

Table 5

Model overview

Model	α	α^+	α^-	η	β
Environment-specific single learning rate model	2	0	0	0	2
Non-environment-specific single learning rate model	1	0	0	0	1
Environment-specific dual learning rate model	0	2	2	0	2
Non-environment-specific dual learning rate model	0	1	1	0	1
Environment-specific variable learning rate model	2	0	0	2	2
Non-environment-specific variable learning rate model	1	0	0	1	1

Note. α is the learning rate; α^+ and α^- are the positive and negative learning rates, respectively; η is the higher-order learning rate; and β is the inverse temperature. 0 indicates that the model (row) does not contain the parameter (column); 1 indicates that the model contains the parameter once, shared across environments; and 2 indicates that the model contains the parameter twice, once for each environment specifically.

In the manuscript, there is no description of the contingencies used in stable and volatile environments nor description of the volatile environment itself. The reader needs those details to fully understand the paradigms and results. For increased clarity, I would recommend to add these details in the main text and not only in the method section.

We added these design details to the results section on page 4:

> "In the stable casino, the slot machine most likely to yield reward (i.e., 80% vs. 20%) remained constant for the duration of a block, while in the volatile casino, the slot machine most likely to yield reward (i.e., 90% vs. 10%) switched every 16-24 trials (within that casino)."

The test phase of the third experiment compounds 200 trials (Figure 1) and reward contingencies switched twice, every 29-37 trials (Methods). Is there a typo in one of these two pieces of information? It is not clear when the switches happen along the 200 trials.

In the test phase of Experiment 3 reward contingencies switched twice in each environment, that is, they switched every 29-37 trials within each environment. We now phrased this more clearly in the manuscript on page 25:

> "That is, in the test phase, in both casinos, the reward rule switched twice (every 29-37 trials within each casino)."

Seeing Q-values in reinforcement learning as beliefs (introduction) is not usual and should be justified. Similarly, "optimal" is used extensively in the manuscript and its use seems inappropriate in many instances. In the abstract, people are described as using "optimal" learning rates, which does not seem to be the case. In the first paragraph of the results section, we can read that an "optimal" learner would use two different learning rates in the environment described, but it is not clear what optimal means for the authors here. In general? In the specific context of the model considered?

We have replaced all references to "beliefs about reward contingencies" with "action values" in the introduction. All references to optimal learning rates have been removed from the manuscript since most participants did indeed not use optimal learning rates. Finally, we have elaborated the sentence referring to "an optimal learner" on page 5 so that it now says:

> "These optimal reward probabilities were determined through reward rate simulations (see Methods for more details), which further established that an optimal learner (behaving according to the Rescorla-Wagner model) would use a different learning rate in each environment to obtain the highest reward rate."

Reviewer 2:

The authors tested whether participants could adjust their learning rates according to environmental cues. Through fitting the models to empirical data from three experiments, the authors consistently found in both the model comparison and the fitted parameters that: 1) the environment-specific learning rate (LR) model was the winning model, and 2) during the

learning phase, learning rates in volatile environments were higher. Based on these modeling results, the authors concluded that people could meta-learn the learning rate.
The modeling results were clear. I believe the main contribution of this paper is associating the adjustment of the learning rate with an environmental cue. In general, the paper's conclusion and results didn't surprise me, but they represented a solid forward step. In my opinion, the most valuable part of this paper is the data; therefore, I suggest the authors make their data open-access after publication. When I'm doing the review, the osf website says that I need to press a "request access" button to apply for access permission, which is not ideal.

Our apologies for this unfortunate oversight. The data had indeed not been made public on OSF yet, but have now been made public.

I feel that the word "meta" is overused in this paper. In the current experiment, all LR adjustments were based on a visual cue (a casino icon), which didn't seem very "meta". More importantly, the authors didn't provide a process model for the environment-specific LR adjustment. Experiments 2 & 3 showed that the LR was changing, especially in the volatile condition. However, the current model didn't provide insight into the normative theory of the LR adjustment at the process level. Removing the "meta" from the title might be better. A related point of interest could be a discussion on how such LR adjustments are achieved by the human brain, both at the algorithm level and the neural implementation level.

It is true that we did not provide a description of the processes underlying the environment-specific regulation of learning rates. Instead, we believe to have presented a new experimental design, that allows for studying the behavioral signatures of meta-learning: participants learned how to learn in each environment, which in this case means setting the hyperparameters learning rate and temperature, with concrete contextual cues allowing for these abstract learning strategies. This said, we now see how our emphasis on the word "meta-learning" in the title could be misleading for people expecting a computational model that details how meta-learning may be implemented. Therefore, we changed the title accordingly to: "Learning environment-specific learning rates."

The authors used model comparison and fitted parameters as two main supports for their conclusions. While a model-based analysis is good and valuable, I wonder if it would be possible to include some model-free analysis of the data. Model-free analysis is valuable because it usually requires fewer mathematical assumptions and can illustrate behavioral phenomena more directly. Figure 6B represents a model-free analysis, but I believe it's insufficient. For example, the authors could try using "whether participants made a mistake in the last trial" as a regressor to predict "whether participants switch their decision in the future trial". After performing the regression, the authors could compare the regression coefficients in different conditions and gain more model-free intuition into the data. A similar analysis was performed in (Vickery et al., 2011).

We agree that these model-free analyses can allow for a more comprehensive and assumption-free understanding of the data, and would like to thank the reviewer for this interesting analysis suggestion. We now added these analyses to our paper. For example, for Experiment 2, one can now read on page 11 that:

> "In the volatile environment participants were more likely than in the stable environment to choose the other slot machine than the one they chose on the last trial (within that environment) if that choice did not yield reward ($t(50) = 3.755$, $p < .001$)."

The results for Experiment 1 (page 9) and Experiment 3 (page 15) were very similar.

Given that Behrens has already conducted a related simulation, I'm not surprised that people should adjust the learning rate in different environments. For me, Figures 1B and 2 don't seem necessary. Moreover, I think the phrase "people can learn to use different, optimal learning rates" in the abstract is an overstatement. The current optimality analysis shows that the direction of the fitted LR is good (i.e., high LR in a volatile environment), but it's difficult to determine whether such LR is optimal. Optimality is a strong claim and should be used cautiously. To make such a claim, the authors might need a different model format (Lieder & Griffiths, 2017, 2020; Sims et al., 2012). A classic form of such a format includes an "argmax" in the model. When I read "Here, using optimality simulations and Bayesian hierarchical

modelling across three experiments, we show that people can learn to use different, optimal learning rates when switching back and forth between two different environments." in the authors' abstract, I initially thought that the authors were modeling the agent as an optimal Bayesian decision maker in a hierarchical environment, but then I found I had misunderstood it. Since "Bayesian hierarchical modeling" is just a fitting technique, removing it could help the reader avoid possible confusion.

Thank you for pointing out our potentially confusing use of these terms. We removed all references to participants using optimal learning rates. We also changed “Bayesian hierarchical modelling” to “hierarchical Bayesian analyses” in the abstract. However, we believe that figures 1B and 2 (now figures 2B, 3B, and 4B) are still valuable, as they demonstrate that our particular design (i.e., number of trials, reward probabilities, and volatilities of reward probabilities in each environment) requires different learning rates in the different environments to maximize reward, and offer a potential explanation for why people might adapt negative learning rates more than positive learning rates. We now also used these same plots to display the variability in individual parameter estimates (as well as their proximity to optimality).

I recommend moving the model validation to the supplementary material for readability. Additionally, did the authors try other model validation methods (e.g., parameter recovery)? Recovering the reward rate doesn't provide strong evidence that the model has a valid fit to the data because the reward rate is a crude behavioral metric.

Reward rates are indeed a crude behavioural measure. Nevertheless, we feel that correlations of .8 and higher between observed and recovered reward rates demonstrate a remarkably good fit of the model to the data. However, we also performed parameter recovery simulations, which can be found in the Methods section, on page 31. We now also added a model recovery analysis, which can also be found in the Methods section, on page 30. All three forms of model validations (reward rate recovery, parameter recovery, and model recovery) suggest a good model fit. We now moved all model validations to the methods section.

Reviewer 3:

In their paper, Simoens and colleagues address one main question with help of three experiments: Do humans meta-learn about the volatility of reward environments and adapt their learning rates accordingly. The meta-learning is tested by randomly exposing participants to different reward environments of different volatility and see whether participants learn with different learning rates on a trial-by-trial basis. Furthermore, they investigated whether participants actually associate different environments with different learning rates (and thus apply them according to the given environment) or whether the different learning rates are just a by-product of a difference in experienced prediction errors.

The paper is well written and uses well-designed experiments and state-of-the-art hierarchical Bayesian modeling to answer the research questions and use parameter recovery validate the fitting procedure. The methods and hypotheses have been pre-registered and deviations are clearly described and motivated. All in all, the manuscript is of high quality and I do not have any major concerns, but rather some additional questions:

Since the experiment was performed online there is much less control over the testing environment. This is also reflected in the high number of participants that had to be excluded because their reward rate did not significantly exceed that of random choice behavior. I thus think it would be important to add some further characterization of behavior for the included participants. This might be things like reaction times or missed trials (maybe for the different blocks to see changes over time), maybe the authors have some other ideas. Also, did all included participants complete all blocks of the respective experiments?

We now provided more information about the inclusion criteria by describing the reaction times and missed trials for the included participants on page 23:

> "In all three experiments, participants were excluded from the analyses if their reward rates were not significantly higher (according to chi-square tests with an alfa-level of 0.05) than chance level in either (or both) of the two environments in the learning phase. This led to the removal of 14 participants in Experiment 1, 12 participants in Experiment 2, and 31 participants in Experiment 3. All participants completed all experimental blocks. A response time deadline of 5 seconds was implemented in all

three experiments, but none of the included participants failed to give a response in more than 4.58% of trials, while 93.04% of the included participants failed to give a response in less than 1% of trials. The mean response time was 677 ms (SD = 411 ms)."

Another interesting measure would be the characterization of how close to optimality participants learn. Maybe you could overlay the simulation plots in Figure 2 with points showing the estimated learning rates for each participant, color-coded in with the reward rate?

We would like to thank the Reviewer for this suggestion. These are indeed highly informative. We now included the suggested figures in the manuscript. For Experiment 2, for example, these figures can be found on page 13:

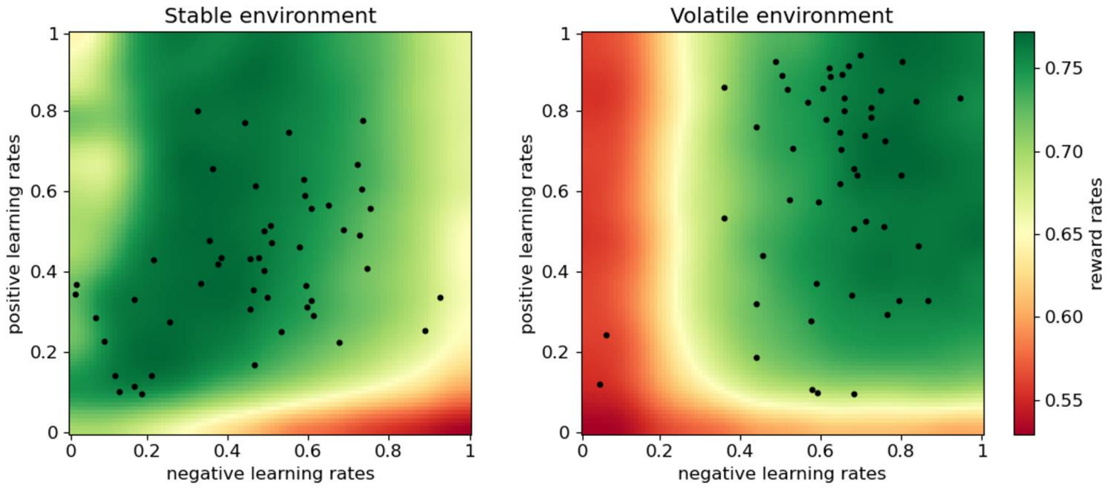

The figures look very similar for Experiment 1 (page 10) and Experiment 3 (page 16).

I am wondering why there is a general tendency to start learning with too low learning rates which then increase over time. You obviously investigate your main question, whether the difference between the learning rates in the stable vs. the volatile environment increases over time. But going beyond that, do you have any idea why the general trend for the learning rates is to increase, i.e. why do you not see participants starting with too high learning rates that decrease towards optimality?

We conjecture that people might have a default learning rate with which they start learning in a novel environment before they have had enough experience to adapt to it. Perhaps this default learning rate is rather low because our everyday world tends to be stable. Alternatively, this could also be due to the framing of our experiment in terms of gambling in a casino, where reward distributions are often thought to be extremely noisy and impossible to learn. We now added these potential explanations for this data pattern to the discussion, on page 21:

> "We observed an increasing difference between the two environment-specific learning rates over blocks, again suggesting that participants gradually learned to optimise and associate different learning rates to both environments. On top of this, however, participants seemed to have a tendency to start learning with rather low learning rates which they then increased across blocks. This is merely conjecture, but people might have a default learning rate with which they start learning in a novel environment before they have had enough experience to adapt to it. Perhaps this default learning rate is rather low because our everyday world tends to be rather stable. Alternatively, this could also be due to the framing of our experiment in terms of gambling in a casino, where reward distributions are often thought to be extremely noisy and impossible to learn."

Looking at the simulation plots in Figure 2 you nicely show that only looking at the positive or the negative learning rate, it is not always possible to say whether a given learning rate is optimal or not, it is the combination of both that really matters for achieving a high reward rate. Therefore, the plots in figures 4b and 5c are difficult to interpret in relation to reward-rate optimization. Could you maybe somehow elucidate this trend more in relation to reward rate optimization?

It is indeed the combination of both positive and negative learning rates that matters for achieving a high reward rate. We tried to shed light on this issue by adding the following paragraph to the manuscript, on page 9:

> “As can be seen in Figure 2B, these simulations showed that also an agent with dual learning rates benefits more from higher learning rates in the volatile than in the stable environment. This effect is more pronounced for negative learning rates (i.e., learning from negative prediction errors). More specifically, in the stable environment, lower negative learning rates tend to result in higher reward rates, while positive learning rates have less effect on reward rate. In the volatile environment, higher negative as well as positive learning rates tend to result in higher reward rates, but this effect is still more pronounced for negative than for positive learning rates.”

This can help interpret figures 4B (now 3B) and 5C (now 4B) in terms of reward optimization. Moreover, we added, for both Experiment 2 and 3, an analysis that demonstrates that participants acquired higher reward rates in the last compared to the first block of the learning phase. For Experiment 2, for example, this analysis can be found on page 14:

> “In line with our reward rate simulations (Figure 3B), which suggest that using higher learning rates in the volatile than in the stable environment yields higher rewards, reward rates were significantly higher in the fourth compared to the first block ($t(50) = 2.239$, $p = .015$).”

This result was very similar for Experiment 3 (page 17). However, the main purpose of these figures is to demonstrate that participants gradually learned to use different learning rates in both environments, which is more important to our hypothesis than their proximity to optimality.
